# Fine-Grained Face Annotation Using Deep Multi-Task CNN

**DOI:** 10.3390/s18082666

**Published:** 2018-08-14

**Authors:** Luigi Celona, Simone Bianco, Raimondo Schettini

**Affiliations:** Department of Informatics, Systems and Communication, University of Milano-Bicocca, viale Sarca, 336 Milano, Italy; bianco@disco.unimib.it (S.B.); schettini@disco.unimib.it (R.S.)

**Keywords:** face analysis, convolutional neural networks, multi-task learning, gender recognition, age group recognition, face attributes’ estimation

## Abstract

We present a multi-task learning-based convolutional neural network (MTL-CNN) able to estimate multiple tags describing face images simultaneously. In total, the model is able to estimate up to 74 different face attributes belonging to three distinct recognition tasks: age group, gender and visual attributes (such as hair color, face shape and the presence of makeup). The proposed model shares all the CNN’s parameters among tasks and deals with task-specific estimation through the introduction of two components: (i) a gating mechanism to control activations’ sharing and to adaptively route them across different face attributes; (ii) a module to post-process the predictions in order to take into account the correlation among face attributes. The model is trained by fusing multiple databases for increasing the number of face attributes that can be estimated and using a center loss for disentangling representations among face attributes in the embedding space. Extensive experiments validate the effectiveness of the proposed approach.

## 1. Introduction

A detailed face annotation might include a wide variety of information such as: demographic attributes (gender, age and ethnicity), visual attributes (e.g., hair style, clothing, face shape) and emotional state (facial expressions). Automatic systems for face annotation have numerous practical applications including user-verification, video surveillance and image retrieval. In the last few decades, many algorithms have been developed to address problems, such as face recognition and verification [1,2], facial expressions recognition [3,4], landmark localization [5] and facial attributes estimation, independently [6,7]. Recently, new approaches involving the use of multi-task learning (MTL) frameworks [8] for dealing with several face tasks have been presented [9,10]. With respect to single-task learning-based methods, where each task is addressed separately, ignoring any correlations between tasks, MTL-based methods enable learning shared representations.

In order to enrich the annotation of face images, in this paper, we employ a multi-task learning framework to train a single convolutional neural network (CNN) in an end-to-end manner for fine-grained face annotation. Moreover, due to the lack of datasets providing a ground-truth for several tasks, we fuse multiple sources of label information to increase the number of attributes that can be estimated.

The contributions of this paper are:

- a model trained for simultaneously estimating up to 74 different attributes describing face images in terms of gender, age group and visual attributes (e.g., hair color, face shape and the presence of makeup);
- a gating mechanism acting as a routing system to encode attribute-specific features, enabling the proposed model to share all the CNN’s parameters among tasks, thus resulting in a lower number of parameters compared to classical multi-task architectures;
- a layer for conditioning predictions in order to take into account the correlation among attributes based on the co-occurrence matrix estimated on training data.

## 2. Related Work

The proposed approach covers gender and age group recognition, face attribute estimation and multi-task learning. We present an overview of the relevant works organized by these topics.

**Gender and age group recognition.** The Adience benchmark consisting of a challenging collection of face images labeled for age and gender has been designed in [11]. The authors also proposed a system involving the use of LBP descriptors for face representation and dropout-SVM for classification. The use of synthetic frontalized face images was demonstrated to be effective for improving gender recognition performance in [12]. In [13], two different convolutional neural networks (CNNs) have been trained from scratch for addressing respectively gender and age group recognition on the Adience benchmark. A hybrid approach combining deep features of a fine-tuned CNN and a linear SVM has been applied in [14] for face gender recognition on the color FERETand the Adience databases. In [15], the Deep EXpectation (DEX) pipeline consisting of a VGG-16 architecture trained on well-aligned images of faces from the large IMDB-WIKIdataset has been proposed for apparent age estimation, and then, it has been tested on Adience images for age group recognition.

**Face attribute estimation.** The first work on the automatic estimation of multiple attributes is the FaceTracer system [16]. This approach involved the use of independent classifiers for 65 different face attributes trained using several hand-crafted features (i.e., image intensities in RGB and HSV color spaces, edge magnitudes, gradient directions) extracted from hand-picked facial regions. More recent approaches leverage deep learning techniques. The pose-aligned networks for deep attribute (PANDA) model [17] exploited a CNN to localize face parts and to extract features from the localized parts. These features have been used to train SVM classifiers for attribute prediction. In [18], a combination of two localization networks (LNETs) and an attribute recognition network (ANET) have been proposed. LNETs have been trained in a weakly-supervised manner; instead, ANET has been pre-trained by classifying massive face identities and then fine-tuned by attributes to extract features that are fed into independent linear support vector machines (SVMs) for the final classification of 40 face attributes. Experiments have been conducted on two large-scale face attribute databases proposed, namely CelebAand LFWA. The training and usage of CNNs for separately estimating face attributes have been performed in [19,20]. In [21], an ensemble of multi-class SO-SVM predictors has been trained on top of deep features extracted from a VGG-16 architecture pre-trained on ImageNet and then fine-tuned on IMDB-WIKI and ChaLearn2015 LAPdatasets for apparent age, gender and smile prediction. Recently, semantic segmentation has been used to guide the attention of the attribute prediction in [7].

**Multi-task learning.** Deep learning approaches have been proven to be well suited for multi-task learning (MTL). Several MTL approaches have been proposed for attribute estimation. A multi-task restricted Boltzmann machine (MT-RBM) has been used in [6] for facial attribute classification. Some works [10,22] categorized the attributes into different groups to take advantage of their mutual relationships. In [22], the authors suggested an auxiliary network on top of the multi-task CNN to further exploit the relationships among the attributes. In [10], the DMTLwas defined as a modified AlexNet with both shared and category-specific sub-branches to model both attribute correlation and attribute heterogeneity.

## 3. Databases

**Figure 1:**
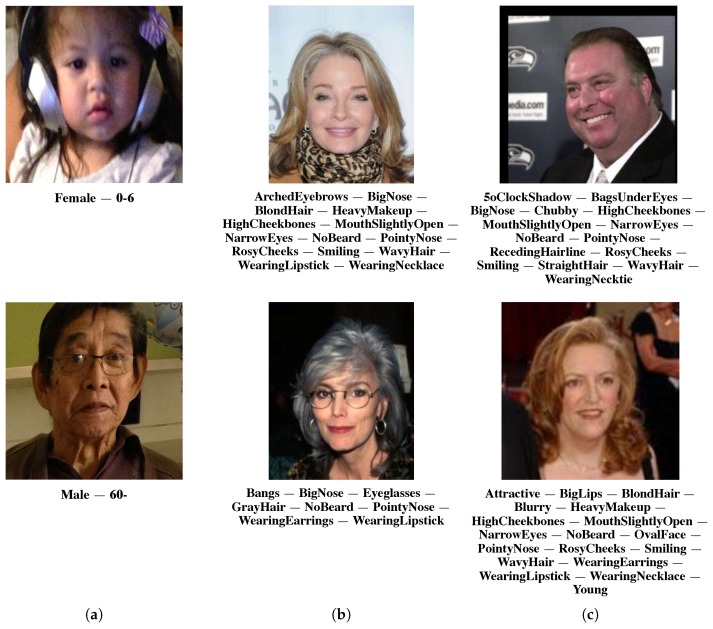
Example images from evaluated databases. (**a**) Adience benchmark image labeled in terms of gender and age group. (**b**) Images from the CelebAdatabase with occurring face attributes coming from the 40 attributes in Table 1. (**c**) Images from the LFWAdatabase and occurring attributes from the same list of 40 attributes as in the CelebA database.

For the evaluation of the proposed end-to-end multi-task convolutional neural network, we selected the three most widely-used publicly available face databases for face attribute estimation (see Figure 1).

**Adience benchmark.** The Adience benchmark [11] is a database designed for age and gender classification. Images were collected from Flickr uploads mainly from smart-phone devices, and they were manually labeled with one among eight age groups (0–2, 4–6, 8–13, 15–20, 25–32, 38–43, 48–53, 60+ years) and gender annotations. Faces from Adience are highly unconstrained, reflecting many of the real-world challenges, such as occlusions, extreme variations in head pose and lighting conditions’ quality. The database contains about 26 K images of 2284 subjects.

**CelebA.** CelebA is a large-scale face attributes database [18] consisting of more than 200 K images of more than 10 K celebrities. Each facial image is manually annotated with 40 binary attributes (see Table 1). Database images present high variations in pose, expression, race, background and imaging conditions. CelebA provides two type of images, i.e., aligned and cropped face images and raw images. For the experiments, we used the aligned and cropped face images.

**LFWA.** LFWA is an unconstrained database for face attributes’ estimation [18]. It has 13,233 images of 5749 identities automatically annotated with the same 40 binary attributes as in the CelebA database (see Table 1). Recently, a larger set of annotations consisting of 73 binary attributes was released (see Table A1 in Appendix A for the complete list). The machine-based ground-truth for the 73 face attributes is integrated in the set of files that can be downloaded from the following website: http://mmlab.ie.cuhk.edu.hk/projects/CelebA.html.

## 4. Deep Multi-Task Learning for Face Annotation

The aim of the proposed approach is to simultaneously estimate a large number of facial attributes using a single model.

Multi-task learning by convolutional neural network models has been demonstrated to be very effective for many face-related tasks [10,23,24]. Following this success, a multi-task learning approach based on a convolutional neural network (MTL-CNN) to estimate multiple facial attributes jointly from a single face image was proposed. This model takes into account the attribute inter-correlations to obtain informative and robust feature representation. This property is desirable for problems like facial attributes estimation, where classes are correlated with each other. As shown in Figure 3, several attributes of the CelebA have strong pair-wise correlations (elements with red color). For example, “Male”, “Attractive” and “NoBeard” are highly correlated; this means that gender the and presence/absence of a beard affect face’s attractiveness.

For an input face image, the resulting model is able to predict all the learned facial attributes jointly. Given that many of the public-domain databases provide a ground-truth only for a subset of desired facial attributes (e.g., only facial expressions or soft biometrics), the training set is composed by aggregating data from multiple databases labeled with a single attribute. The MTL-CNN consists of shared parameters for all the tasks, while parameters from lower layers adapt to the complete set of domains, and attribute-specific parameters are specialized for the estimation of each attribute. The aforementioned CNN is trained by combining two different losses: the first is used for attributes that are defined as mutually exclusive (e.g., age group and gender); instead, the second is used for attributes that are defined as co-occurrent. Additionally, a gating mechanism is introduced in order to pass/suppress information. Finally, to better reflect the correlation between facial attributes, a label post-processing layer is applied.

### 4.1. The Model

**Figure 2:**
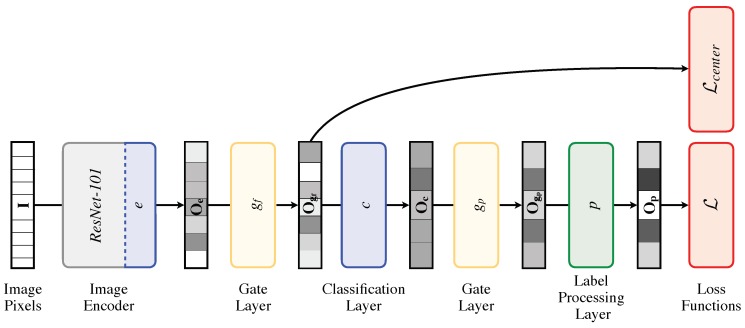
A complete illustration of our model for face annotation. The face image **I** is fed into the network. The model consists of seven components: the image encoder (*e*), which produces the output $O_{e}$ by a non-linear transformation of the activations of ResNet-101; the gate layer ($g_{f}$) designed to remember or forget features across different attributes and that produces the output $O_{g_{f}}$; the classification layer (*c*), which produces the output $O_{c}$, i.e., the logits for the considered attributes; the gate layer ($g_{p}$) designed to remember or forget probabilities across different attributes and that produces the output $O_{g_{p}}$; the label processing layer (*p*) designed to reflect the relationships among the different attributes and that produces the output $O_{p}$; and the loss functions (L and $L_{center}$).

Figure 2 shows our architecture for face annotation. We first introduce the CNN architecture we used for encoding face images and then explain the other components in the following sections.

#### 4.1.1. Image Encoder

ResNet-101 [25] is used as the CNN architecture for the image encoder. It has 101 parameter layers: the first is a 7×7 convolutional layer followed by four blocks each containing 3, 4, 23, 3 residual units, respectively. The network ends with a global average pooling layer followed by a fully-connected layer. This last layer represents the classification layer, which directly maps the output of the average pooling layer into logits. To obtain global features related to different face regions, we replace the classification layer with $e:R^{2048}\mapsto R^{2048}$, which consists of a fully-connected layer followed by ReLU non-linearity and producing $O_{e}\in R^{2048}$ as a feature vector.

#### 4.1.2. Gating Mechanism

Gate layers are employed to select contextual attribute features. The idea is based on the mechanism of the gate unit in Long Short-Term Memory (LSTM) [26], which is used to learn to remember or forget the history information from a long sequence of input data. Differently from LSTM, the introduced gate layers do not depend on temporal data, but are designed to “remember” or “forget” features across different attributes. The gate equation used is:

(1) $$\begin{array}{c} y=\sigma (W_{g}x+b)\cdot x, \end{array}$$

where σ is the element-wise sigmoid non-linearity, $W_{g}$ and b are learnable parameters, x is a feature vector and (·) indicates the element-wise multiplication. The gate function is used at the feature-level and prediction-level. The feature-level gate, $g_{f}:R^{2048}\mapsto R^{2048}$, defined as follows:

(2) $$\begin{array}{c} O_{g_{f}}=\sigma \left(W_{g_{f}}O_{e}+b_{g_{f}}\right)\cdot O_{e}, \end{array}$$

is introduced immediately before the classification layer to disentangle the representation of the different attributes. The prediction-level gate, $g_{p}:R^{48}\mapsto R^{48}$, controls the co-occurrence between predicted attributes, and it is formalized as follows:

(3) $$\begin{array}{c} O_{g_{p}}=\sigma \left(W_{g_{p}}O_{c}+b_{g_{p}}\right)\cdot O_{c}. \end{array}$$

#### 4.1.3. Classification Layer

The classification layer is defined as $c:R^{2048}\mapsto R^{48}$ and consists of a fully-connected layer. It takes $O_{g_{f}}$ as the input and outputs the 48 logits $O_{c}$ for the 40 binary attributes plus the eight age groups into which the age attribute is divided.

#### 4.1.4. Label Processing Layer

**Figure 3:**
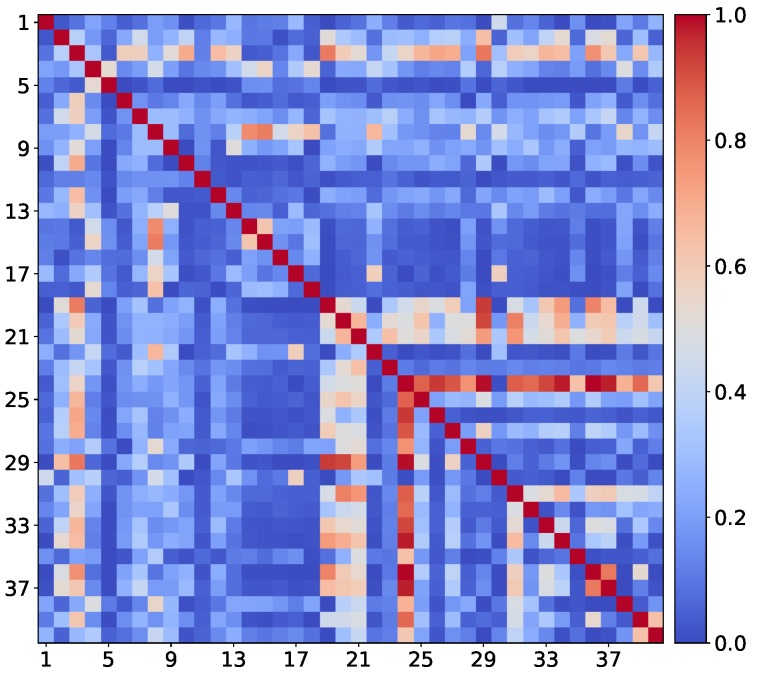
Co-occurrence matrix of the 40 attributes provided with the CelebA database (only training set labels are considered). Blue means low correlation among face attributes, while red means high correlation among face attributes.

The label processing layer is designed to reflect relationships among facial attributes. For example, in the CelebA database [18], “WearingLipstick”, “RosyCheeks” and “HeavyMakeup” are strongly correlated. Although some relationships among face attributes might be peculiar to this specific data distribution [27,28], in order to exploit this information, the co-occurrence matrix $M_{c}$ (see Figure 3) is computed by counting the number of pair-wise co-occurrences for the 40 facial attributes. The co-occurrent value $M_{c}\left[i,j\right]$ between the *i*-th and the *j*-th attribute is calculated as higher as *i*-th and *j*-th appear together in more images. Label processing layer *p* is then formalized as follows:

(4) $$\begin{array}{c} O_{p}=ReLU\left(W_{\mathrm{lp}}M_{c}O_{g_{p}}\right)\cdot O_{g_{p}}. \end{array}$$

Here, ReLU is the element-wise ReLU non-linearity; $M_{c}$ is the co-occurrence matrix; $W_{\mathrm{lp}}$ is a weights matrix; $O_{g_{p}}$ is the prediction; and (·) indicates the element-wise multiplication. The label processing layer multiplies the original prediction $O_{g_{p}}$ by its processed version in order to weight the original prediction and not to replace it.

Whether the predicted probability $o_{Male}$ is around one, while probabilities $o_{HeavyMakeup}$ and $o_{WearingLipstick}$ are high, given that the pair-wise correlation between these attributes is high, the resulting probability for the attribute “Male” due to the label processing layer will be penalized. Differently from the prediction-level gate, which learns the relationships among attributes in a self-supervised manner (see Section 4.1.2), the label processing layer exploits the co-occurrence matrix to find out attributes with strong correlation.

#### 4.1.5. Mutually Exclusive vs. Co-Occurrent Attributes

Many of the considered attributes are mutually exclusive, i.e., only one class is the correct one. For example, for the age group problem, it is not possible that the same subject is simultaneously classified in the ranges “15–20” and “25–32”. For these attributes, softmax cross-entropy loss, the most popular loss function for single-label image classification in CNNs, is used. On the other hand, co-occurrent attributes are present such as “Smiling” and “Mustache”. These are attributes that can simultaneously occur, and practically, this means that their probabilities are independent. The binary cross-entropy loss is used because, unlike the softmax, which gives a probability distribution around classes, it allows one to deal with multi-label problems. Although the CelebA and LFWA databases contain facial attributes that are mutually exclusive such as “BrownHair”, “BlackHair” and “BlondeHair” and the use of softmax cross-entropy would enhance the learning algorithm to maximize only one attribute among them, given that the ground-truth says only the presence/absence of the attribute, the estimation of these attributes is maintained as a multi-label problem (instead of a single-label multi-class problem). The algorithm would adapt to learn such dependencies.

#### 4.1.6. Center Loss

Center loss was first proposed for the face recognition task [29] and used for other problems because of its effectiveness at making discriminative embedding features. The purpose of center loss is to minimize intra-class variations while maximizing inter-class variations. The original center loss was designed for the single-label classification problem, and it is hard to exploit for multi-label classification. Therefore, we modified the criterion in order to address the multi-label classification problem as follows:

(5) $$L_{Center}=\frac{1}{M}\sum_{i=0}^{M}\left(\frac{1}{N}\sum_{j=0}^{N}∥O_{g_{f}}^{\left(i\right)}-c_{y_{j}}^{\left(i\right)}{∥}_{2}^{2}\right),$$

where $O_{g_{f}}^{\left(i\right)}$ is the embedding feature vector from the feature-level gate layer for the *i*-th sample, $c_{y_{j}}^{\left(i\right)}$ is the class center feature vector (centroid) for the corresponding ground-truth label and *M* is the mini-batch size. More in detail, the centroids’ matrix is defined as N×2048, where *N* is the number of attributes. Each embedding feature vector $O_{g_{f}}$ is compared with all the centroids corresponding to 1 s in $y_{j}$, which is one-hot encoded.

### 4.2. Training

The model is trained end-to-end by using random batches of images: half of the samples are taken from the Adience benchmark, and the remaining half are taken from the CelebA database. We perform data augmentation by producing a crop with a random size covering at least 80% of the original image and a random aspect ratio of 3/4–4/3 of the original aspect ratio. The obtained crop is finally resized to the given size of 224×224 pixels. Furthermore, we obtain diverse samples by horizontally flipping the input image and, finally, by applying color jittering (brightness and contrast). We train our MTL-CNN using SGDwith Nesterov momentum, using a batch size of 32 samples, and the learning rate is kept fixed to a value of $1\times 10^{-4}$. Momentum is set to 0.9, and the weight-decay parameter is $5\times 10^{-4}$. The model is trained for 15 epochs, and the best model is selected using the early stopping strategy (i.e., the best performance achieved in all tasks on the validation sets). The total loss used to train the model is given by $L_{total}=L_{CE}+L_{BCE}+\alpha L_{center}$, where $L_{CE}$ is the softmax cross-entropy loss, $L_{BCE}$ is the binary cross-entropy, $L_{center}$ indicates the center loss and α weights the contribution of the center loss and has been heuristically set to 0.95.

## 5. Experiments and Results

In the following, we detail the experiments conducted and the evaluation protocols, and we compare the performance of the proposed model with state-of-the-art methods.

Experiments were conducted by simultaneously training the model on two databases: the Adience benchmark and one among the CelebA and the LFWA (see Section 3). The test sets of the corresponding datasets were used for evaluation. Moreover, to supplement the analysis and to demonstrate the generality of the proposed method, the experimental results were also collected in a cross-database fashion.

### 5.1. Evaluation Protocols

Testing for both age group and gender classification on the Adience benchmark was performed using the standard five-fold, subject-exclusive cross-validation protocol defined in [13], while for the CelebA and LFWA databases, the results were computed on the test set provided in [18]. Specifically, the average accuracy over the five-folds of cross-validation is reported for both age and gender classification; concerning facial attributes’ estimation on the CelebA and LFWA databases, the average classification error on the test set obtained by the five models resulting from the cross-validation is reported.

### 5.2. Experimental Results

Since the source codes are not available for any of the other state-of-the-art methods, we report the results directly from the corresponding publications.

**Gender and age group recognition:** Results for gender and age group classification on the Adience benchmark are measured in terms of accuracy in Table 2. For age group classification, both the accuracy when the algorithm gives the exact age group classification and when the algorithm is off by one adjacent age group (i.e., the subject belongs to the group immediately older or immediately younger than the predicted group) were measured. For gender classification, we achieved a performance of 90.7%, which was around 3% higher than the best result in the state-of-the-art [14].

For the age group task, the obtained accuracy corresponded to 57.8% for exact classification and 94.1% for 1-off classification. This performance was lower than those of RAGN [30] and DEX [15] for both exact and 1-off classification accuracy. These two approaches took advantage of the fine-tuning of a pre-trained CNN on the large IMDB-WIKI database: in fact, the results of and end-to-end learning of DEX dropped respectively to 55.6±6.1 for exact and 89.7±1.8 for 1-off classifications. The pre-training on IMDB-WIKI improved the model generalizability, and this is very important especially given that samples in folders were not homogeneously divided. This was confirmed by the huge difference among classification accuracy obtained for distinct folders: 68.3% on the first folder, while 49.72% on the second one. When predicting the 1-off accuracy, we achieved 94.1%, which was 2% lower than DEX with IMDB-WIKI pre-training and 4% higher than DEX without IMDB-WIKI pre-training.

**Face attribute estimation.** The experimental results for CelebA and LFWA databases are expressed in terms of classification error. The performance of the proposed method is compared with state-of-the-art methods for CelebA and LFWA databases in Table 3. The column “Prior” shows errors obtainable by only applying the knowledge from the prior probabilities of the presence or absence of an attribute in the datasets. The first four rows report results for 40 binary attributes’ estimation. More in detail, the first row shows the classification error for the CelebA test set classified using a model trained on the CelebA training set, and it corresponds to 8.69%. The third row shows the classification error for the LFWA test set classified using a model trained on the LFWA training set, and it is equal to 15.59%.

Cross-database testing results are reported in Rows 2 and 4; 26.40% and 23.33% were the errors on the CelebA and LFWA test sets, respectively. These errors were lower than the ones obtained by DMTL [10], which was the only one dealing with this testing setup. This indicates that the proposed algorithm generalized better than other methods and that it was more appropriate to apply in the wild.

**Figure 4:**
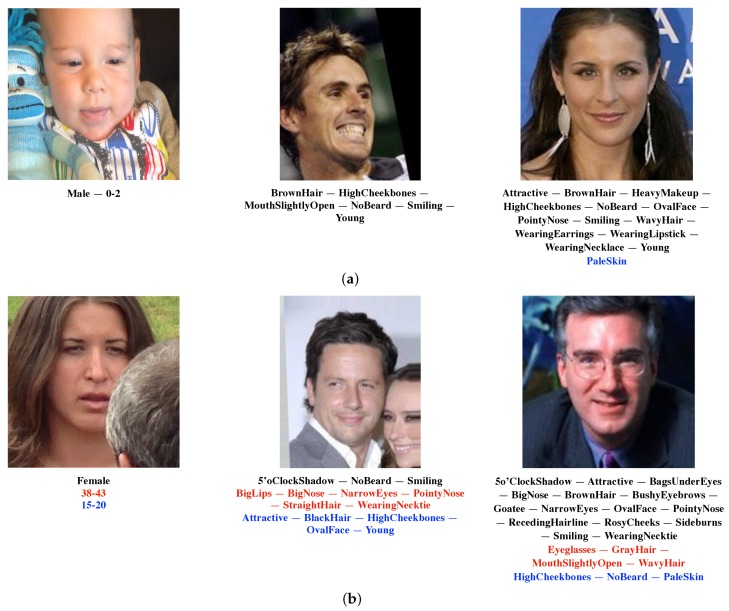
Some sample images annotated using the proposed model. Correct (black), missed (red) and wrong (blue) face attributes are reported. (**a**) Correctly-annotated samples (from left to right: Adience, CelebA and LFWA). (**b**) Samples wrongly annotated (from left to right: Adience, CelebA, and LFWA).

The performance for the larger set of 73 annotations is reported in the last row of Table 3. This was the first time results had been collected for these labels, so there was no comparison with the state-of-the-art methods. The error percentage was considerably smaller than the one achievable by only using the prior probabilities; furthermore, it was even lower than the one obtained for 40 attributes’ estimation. Figure 4 shows some sample images annotated using the proposed model.

### 5.3. Ablation Study

The core ideas of the proposed model lie in the gating mechanism, the module to post-process the predictions, namely the label processing layer, and the center loss. In this subsection, we evaluate the contribution they provided to the final model.

#### 5.3.1. Gating Mechanism

The purpose of the gating mechanism and specifically of the feature-level and prediction-level gate layers was to disentangle the representation among different attributes. The entries denoted with noGates in Table 2 report the performance of the model without the use of gates, respectively for gender (Table 2a) and age group (Table 2b) recognition. Testing results showed a lower performance with respect to the final model of around 3% for the gender recognition task and of around 5% for the age group recognition task. Column noGates in Table 3 shows the performance of the model without gates for face attributes’ estimation. Also for this task, the model without gates performed worse than the final one. Comparing the improvement given by the gate layer across the three tasks, we can observe that the smallest improvement was gained for the gender recognition task. This difference could be motivated by the fact that features for age group and face attributes’ estimation was required to be more discriminative than the ones for gender recognition. The importance of the gating mechanism is further supported by the huge improvement obtained by adding it in the final model trained on the larger set of 73 face attributes.

#### 5.3.2. Label Processing

To evaluate the importance of the Label Processing (LP) layer, we estimate the performance also for a model without this specific layer. The column noLP in Table 3 reports the classification error for all the experiments we ran. Given that the LP layer influenced only attributes’ estimation, we do not report any results for the tasks of gender and age group recognition. From the results, we see that the model with the LP layer performed better than the one using only the two gating layers.

#### 5.3.3. Center Loss

The importance of using the center loss as an additional criterion during the training is evaluated in this subsection. Specifically, we trained the model comprising both gate and label processing layers without using the center loss. The entries for noCenter in Table 2 show the performance of the model trained without the use of the center loss, respectively for gender (Table 2a) and age group recognition (Table 2b), while Column Table 3 reports the results of the proposed model trained without center loss for face attribute estimation. We can observe that the performance obtained by the model trained without the use of the center loss was worse than the final one. This supports the importance of using the center loss both for regularizing the training and for increasing the discriminative power of the features.

## 6. Conclusions

In this work, we have made the following contributions: First, we have designed a multi-task model able to simultaneously predict up to 74 tags describing face images. While other CNN-based methods used sub-branches towards individual attribute estimation, the proposed model shared all the CNN’s parameters. One of the key ideas was to focus on the use of mechanisms that could manage the flow of information among attributes. To this end, we proposed two architecture components: a gating mechanism to route attribute-specific feature encoding and a layer for conditioning predictions in order to take into account the correlation among attributes. We also defined a new loss function based on the center loss to disentangle attributes’ representation in the embedding space by learning a distinct center for each attribute. Experimental results demonstrated the effectiveness of the proposed model. Moreover, cross-dataset results highlighted the robustness of the approach. Since learning on different classes of visually similar tasks pushes representation to be more discriminative, we assume the proposed model stores knowledge useful for solving different, but related problems.

## Figures and Tables

**Table 1:** List of the 40 face attributes provided with the CelebA database.

Index	Definition	Index	Definition	Index	Definition	Index	Definition
1	5o’ClockShadow	11	Blurry	21	Male	31	Sideburns
2	ArchedEyebrows	12	BrownHair	22	MouthSlightlyOpen	32	Smiling
3	Attractive	13	BushyEyebrows	23	Mustache	33	StraightHair
4	BagsUnderEyes	14	Chubby	24	NarrowEyes	34	WavyHair
5	Bald	15	DoubleChin	25	NoBeard	35	WearingEarrings
6	Bangs	16	Eyeglasses	26	OvalFace	36	WearingHat
7	BigLips	17	Goatee	27	PaleSkin	37	WearingLipstick
8	BigNose	18	GrayHair	28	PointyNose	38	WearingNecklace
9	BlackHair	19	HeavyMakeup	29	RecedingHairline	39	WearingNecktie
10	BlondHair	20	HighCheekbones	30	RosyCheeks	40	Young

**Table 2:** Gender and age group recognition results on the Adience benchmark. Mean accuracy ± the standard error over all ages is reported (a). For age group recognition (b), we measure the accuracy both when the algorithm gives the exact age group (exact) and when the algorithm is off by one adjacent age group (1-off).

(a)		(b)		
Method	Accuracy	Method	Exact	1-off
Eidinger et al. [11]	77.8±1.3	Eidinger et al. [11]	45.1±2.6	79.5±1.4
Hassner et al. [12]	79.3±0.0	Levi et al. [13]	50.7±5.1	84.7±2.2
Levi et al. [13]	86.8±1.4	DEX [15]	64.0±4.2	96.6±0.9
van de Wolfshaar et al. [14]	87.2±0.7	RAGN [30]	66.5±5.1	–
Proposed (noGates)	87.9±0.9	Proposed (noGates)	52.9±4.3	91.3±1.5
Proposed (noCenter)	89.8±1.8	Proposed (noCenter)	56.4±5.8	92.6±1.6
Proposed	90.7±1.7	Proposed	57.8±6.4	94.1±1.6

**Table 3:** Binary attribute estimation results on the CelebA and LFWA databases. The performance was evaluated by classification error on the LFWA and CelebA. Results are reported both for the set of 40 binary attributes and for the new larger set of 73 attributes.

Training	Testing	#attr.	Classification Error (%)									
			Prior	[16]	[17]	[18]	[7]	[10]	Proposed	Proposed	Proposed	Proposed
									(noGates)	(noLP)	(noCenter)	
CelebA	CelebA	40	19.43	18.88	15.00	12.70	8.20	7.00	12.20	9.28	9.27	8.69
CelebA	LFWA	40	–	–	–	–	–	27.00	31.12	26.95	26.93	26.40
LFWA	LFWA	40	28.73	26.07	19.00	16.00	12.87	16.08	18.02	16.00	15.96	15.59
LFWA	CelebA	40	–	–	–	–	–	29.80	26.84	23.94	23.83	23.33
LFWA	LFWA	73	35.18	–	–	–	–	–	18.24	15.62	14.15	13.31

**Figure A1:**
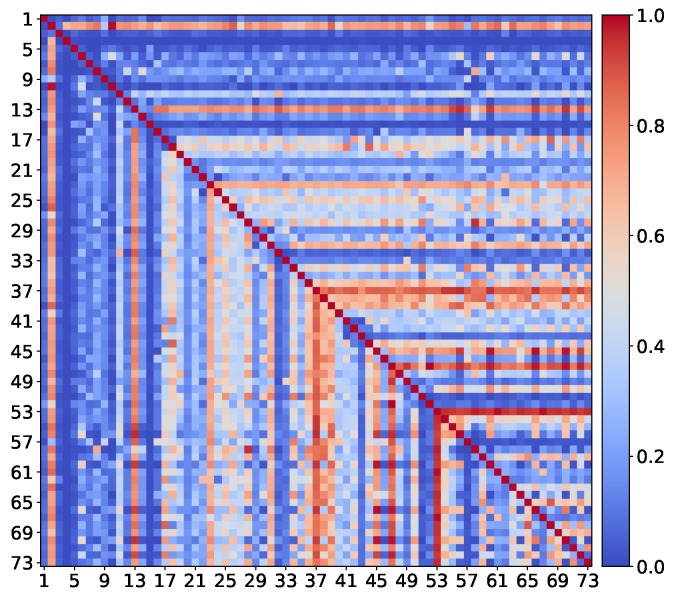
Co-occurrence matrix of the 73 attributes provided with the LFWA database (only training set labels are considered).

## Appendix A

In this Appendix, we report the extended list of 73 face attributes for the LFWA database (see Table A1) and the co-occurrence matrix computed by counting the number of pair-wise co-occurrences for the same set of attributes in Figure A1.

**Table A1:** List of the 73 face attributes recently released for the LFWA database. Overlapping attributes with the smaller list of 40 are in bold.

Index	Definition	Index	Definition	Index	Definition	Index	Definition
1	Asian	21	HarshLighting	41	MouthClosed	61	**RosyCheeks**
2	White	22	Flash	42	**MouthSlightlyOpen**	62	ShinySkin
3	Black	23	SoftLighting	43	MouthWideOpen	63	**PaleSkin**
4	Baby	24	Outdoor	44	TeethNotVisible	64	**5o’ClockShadow**
5	Child	25	CurlyHair	45	**NoBeard**	65	StrongNose-MouthLines
6	Youth	26	**WavyHair**	46	**Goatee**	66	**WearingLipstick**
7	MiddleAged	27	**StraightHair**	47	RoundJaw	67	FlushedFace
8	Senior	28	**RecedingHairline**	48	**DoubleChin**	68	**HighCheekbones**
9	**BlackHair**	29	**Bangs**	49	**WearingHat**	69	BrownEyes
10	**BlondHair**	30	**Sideburns**	50	**OvalFace**	70	**WearingEarrings**
11	**BrownHair**	31	FullyVisibleForehead	51	SquareFace	71	**WearingNecktie**
12	**Bald**	32	PartiallyVisibleForehead	52	RoundFace	72	**WearingNecklace**
13	NoEyewear	33	ObstructedForehead	53	ColorPhoto	73	**Male**
14	**Eyeglasses**	34	**BushyEyebrows**	54	PosedPhoto		
15	Sunglasses	35	**ArchedEyebrows**	55	AttractiveMan		
16	**Mustache**	36	**NarrowEyes**	56	AttractiveWoman		
17	**Smiling**	37	EyesOpen	57	Indian		
18	Frowning	38	**BigNose**	58	**GrayHair**		
19	**Chubby**	39	**PointyNose**	59	**BagsUnderEyes**		
20	**Blurry**	40	**BigLips**	60	**HeavyMakeup**		
